# Clinical Profile, Risk Factors, and Antimicrobial Resistance Patterns of Ventilator-Associated Pneumonia Due to Acinetobacter baumannii in the ICUs of a Tertiary Care Centre in South India

**DOI:** 10.7759/cureus.112059

**Published:** 2026-07-04

**Authors:** Saad Siddiqui, Jayaprakash Appajigol, Preeti Maste, Naveen Angadi, Isha Desai, Viharkumar R Dhorajia

**Affiliations:** 1 General Medicine, KLE Academy of Higher Education and Research (KAHER), Jawaharlal Nehru Medical College, Belagavi, IND; 2 Microbiology, KLE Academy of Higher Education and Research (KAHER), Jawaharlal Nehru Medical College, Belagavi, IND

**Keywords:** acinetobacter baumannii, antibiotic resistance, colistin sensitivity, intensive care unit, mortality prediction, multidrug-resistant organisms, pan-drug resistance, prognostic scores, saps ii, ventilator-associated pneumonia

## Abstract

Introduction

Ventilator-associated pneumonia (VAP) is a major cause of morbidity and mortality in ICU settings, with a greater impact when caused by multidrug-resistant *Acinetobacter baumannii*. This study examines the clinical profile, risk factors, antimicrobial resistance patterns, and prognostic indicators associated with VAP due to this organism in a tertiary care centre in South India.

Methods

This one-year cross-sectional study was conducted from January to December 2023 at KLE’s Dr. Prabhakar Kore Hospital, Belagavi. A total of 68 patients with culture-confirmed VAP due to *Acinetobacter baumannii* were included. Clinical characteristics, comorbidities, risk factors, microbiological susceptibility, and prognostic scores, including the Glasgow Coma Scale (GCS), Acute Physiology and Chronic Health Evaluation II (APACHE II), quick Sequential Organ Failure Assessment (qSOFA), and Simplified Acute Physiology Score II (SAPS II), were analysed. Antimicrobial susceptibility testing was performed using institutional protocols, and resistance patterns were defined based on susceptibility across antimicrobial classes. Ethical approval was obtained from the institutional ethics committee.

Results

The mean age was 48 years, with 54 males (79.4%). Cerebrovascular accident and sepsis were the most common underlying diagnoses, observed in 22 (32.4%) and 13 (19.1%) patients, respectively. Mechanical ventilation was present in all patients. Central venous catheter use and glucocorticoid therapy were noted in 47 (69.1%) and 37 (54.4%) patients, respectively. Mortality was observed in 44 patients (64.7%). Non-survivors had higher prognostic scores. GCS ≤8, APACHE II >20, qSOFA >1, and SAPS II >43 were associated with higher mortality, with area under the curve (AUC) values of 0.928, 0.954, 0.937, and 0.971, respectively. Pan-drug resistance was observed in 49 (72.1%) isolates, while colistin sensitivity was seen in 11 (16.2%).

Conclusion

VAP due to *Acinetobacter baumannii* in this setting was associated with high mortality and a substantial burden of antimicrobial resistance. Prognostic scores, particularly SAPS II, showed strong associations with mortality; however, these findings should be interpreted in the context of the observational design. The results are hypothesis-generating and require validation in larger multicentre studies.

## Introduction

Ventilator-associated pneumonia (VAP) remains a major cause of morbidity in ICUs, particularly in patients who require prolonged mechanical ventilation. It is defined as pneumonia that develops at least 48 hours after endotracheal intubation or tracheostomy in patients without evidence of pneumonia at the time of airway instrumentation [1,2]. Clinically, it is classified by timing. Early-onset infection that occurs within the first 4 days of ventilation is usually caused by antibiotic-sensitive organisms, whereas late-onset infection that develops after 5 days is more often associated with multidrug-resistant pathogens [2].

Guidelines from the Infectious Diseases Society of America and the American Thoracic Society recommend diagnosing VAP when at least two clinical features are present. These include fever of 38°C or higher, leukocytosis of 12,000 cells per microlitre or more, or purulent respiratory secretions, along with a new or progressive infiltrate on chest radiography [2]. Despite advances in intensive care practice, VAP remains the most frequent healthcare-associated infection among mechanically ventilated patients. Incidence across Asian countries ranges from 3.5 to 46 episodes per 1,000 ventilator days, while data from the International Nosocomial Infection Control Consortium report a global rate of approximately 13.6 per 1,000 ventilator days [3,4].

The microbial pattern of VAP is shaped by several factors, including duration of ventilation, prior antimicrobial exposure, local pathogen prevalence, and the quality of infection control practices. Common Gram-negative organisms include *Pseudomonas aeruginosa*, *Escherichia coli*, *Klebsiella pneumoniae*, and *Acinetobacter* species. *Staphylococcus aureus* is the predominant Gram-positive pathogen [4-7].

Among these organisms, *Acinetobacter baumannii* has emerged as a particularly important pathogen in critical care units. It was initially regarded as a low-virulence organism sensitive to most antimicrobials [8]. Over time, it has developed the ability to survive in adverse environmental conditions, form biofilms, and acquire a wide range of resistance mechanisms [9,10]. These characteristics have led to its persistent presence in ICUs and to its recognition as a high-priority pathogen [10].

Treatment of *Acinetobacter baumannii* infection has become increasingly difficult. Imipenem was once considered the main therapeutic option, but resistance has risen substantially [11]. Empirical therapy in settings where multidrug-resistant organisms are suspected often requires combination regimens that include a beta-lactam or carbapenem with a fluoroquinolone or an aminoglycoside [2]. As resistance has expanded, clinicians have resorted to older drugs such as colistin and other polymyxins despite concerns regarding nephrotoxicity, neurotoxicity, and poor lung penetration. Reports of emerging resistance to colistin have further limited effective options [9,11-14].

Delayed recognition and inappropriate empirical therapy for *Acinetobacter baumannii* VAP are associated with higher mortality, prolonged hospital stay, and greater overall healthcare expenditure [13]. General risk factors for VAP include advanced age, pre-existing illness, invasive procedures, and prolonged hospitalisation [2,7]. The risk of infection with multidrug-resistant *Acinetobacter baumannii *rises with extended intensive care stay, prior antimicrobial exposure, and severe underlying disease, especially in regions where colonisation with resistant strains is common [14,15].

This study was undertaken to evaluate the clinical profile, risk factors, antimicrobial resistance patterns, and outcomes of VAP due to *Acinetobacter baumannii* in a tertiary intensive care setting. While these aspects have been described individually in prior literature, there are limited data examining them together within a unified framework, particularly in settings with a high burden of antimicrobial resistance.

The present study adopts an integrated approach by analysing clinical characteristics, resistance patterns, patient outcomes, and the performance of commonly used prognostic scoring systems within the same cohort. This provides a more comprehensive understanding of disease behaviour in a real-world intensive care environment, especially in an Indian setting where antimicrobial resistance patterns and healthcare dynamics may differ from those reported in existing studies.

## Materials and methods

Study setting and design

This cross-sectional observational study was conducted in the ICU of KLE’s Dr. Prabhakar Kore Hospital and Medical Research Centre in Belagavi. The study focused on adults who developed VAP due to *Acinetobacter baumannii* during the study period.

Study period

Data were collected from January 2023 to December 2023.

Participants

Patients aged 18 years and older who fulfilled the diagnostic criteria for VAP and had a culture-positive isolate of *Acinetobacter baumannii* from blood, tracheobronchial secretions, or bronchoalveolar lavage were assessed for eligibility.

Inclusion criteria

Adults aged 18 years and above, patients with microbiological confirmation of *Acinetobacter baumannii* from the relevant clinical samples, and patients who met the clinical and radiological criteria for VAP were included.

Exclusion criteria

Patients with clinical or radiological evidence of pneumonia prior to endotracheal intubation and those without microbiological confirmation of *Acinetobacter baumannii* were excluded.

Sample size determination

A prevalence of 4.2% for VAP due to *Acinetobacter baumannii *among ICU patients was used for sample size estimation. This was based on the study by Chaari A et al., in which 92 of 2,197 ICU patients fulfilled the criteria for *Acinetobacter baumannii* VAP, corresponding to a prevalence of approximately 4.2% [16].

Sample size was calculated using the single-proportion formula:

n = Z² × p × (1 − p) / d²

where Z = 1.96 at 95% confidence level, p = 0.042, and d = 0.05.

Substituting these values:

n = (1.96)² × 0.042 × 0.958 / (0.05)²

n = 61.84

The minimum required sample size was therefore rounded to 62 patients. After adding a 10% allowance for incomplete data, the final sample size was 68 patients.

Study protocol

Eligible patients were enrolled after obtaining informed consent. Ethical approval for the study was obtained from the Institutional Ethics Committee of Jawaharlal Nehru Medical College, Belagavi (Approval No: MDC/JNMCIEC/115). The study was conducted in accordance with institutional ethical standards.

The microbiology laboratory notified the clinical team of all positive *Acinetobacter baumannii* isolates. Each isolate underwent antimicrobial susceptibility testing according to institutional protocols. Clinical information, comorbid conditions, risk factors, laboratory values, and severity scores were extracted from medical records.

Data collection

Comorbidities, including type 2 diabetes mellitus, hypertension, ischaemic heart disease, and chronic obstructive pulmonary disease, were recorded. Additional risk factors, such as prior surgery, recent antibiotic exposure, smoking, alcohol use, malignancy, prior intubation, and nutritional support modality, were documented. Laboratory parameters, including arterial blood gas values, haemoglobin, total leucocyte count, platelet count, blood urea nitrogen, and serum creatinine, were obtained. Severity scores calculated at diagnosis included the Glasgow Coma Scale (GCS) [17], Acute Physiology and Chronic Health Evaluation II (APACHE II) [18], quick Sequential Organ Failure Assessment (qSOFA) [19], and Simplified Acute Physiology Score II (SAPS II) [20].

Microbiological assessment

*Acinetobacter baumannii* isolates were identified using standard microbiological techniques in the institutional laboratory. All samples were processed using uniform microbiological protocols, and standard diagnostic criteria were applied consistently across sample types.

Antimicrobial susceptibility testing and resistance definitions

Antimicrobial susceptibility testing was performed using institutional laboratory protocols in accordance with standard microbiological practices. The antibiotic panel included beta-lactams, carbapenems, aminoglycosides, fluoroquinolones, tetracyclines, and polymyxins, including colistin.

Resistance patterns were interpreted based on susceptibility results across these antimicrobial classes. Multidrug resistance was defined as non-susceptibility to at least one agent in three or more antimicrobial classes. Pan-drug resistance was defined as non-susceptibility to all antimicrobial agents tested within the institutional susceptibility panel, including colistin.

Statistical analysis

Data were compiled and analysed using the SPSS version 21.0. Continuous variables were expressed as mean ± SD, and categorical variables as number (percentage). Analyses were performed using available complete data.

Associations between categorical variables were assessed using the chi-square test. Unadjusted odds ratios with 95% CIs were calculated to evaluate associations between risk factors and in-hospital mortality.

Receiver operating characteristic (ROC) curve analysis was performed to assess the discriminatory performance of prognostic scores, including GCS, APACHE II, qSOFA, and SAPS II. The area under the curve (AUC), sensitivity, specificity, positive predictive value, and negative predictive value were calculated. AUC values are presented as point estimates without confidence intervals.

Continuous variables were summarised descriptively and were not subjected to inferential statistical comparison. All analyses were based on unadjusted associations without multivariable modelling. A p-value below 0.05 was considered statistically significant.

## Results

Demographic and clinical characteristics

A total of 68 patients with VAP due to *Acinetobacter baumannii* were included. The mean age was 48.32 ± 17.8 years, with ages ranging from 18 to 83 years. Men formed the majority of the cohort, accounting for 54 patients (79.4%), as detailed in Table 1.

**Table 1:** Baseline demographic and clinical characteristics of patients with ventilator-associated pneumonia due to Acinetobacter baumannii. Continuous variables are expressed as mean ± SD, and categorical variables are expressed as number (percentage).

Characteristic	Value
Total patients	68
Age (years), mean ± SD	48.32 ± 17.8
Age range (years)	18 to 83
Sex	
Male	54 (79.4%)
Female	14 (20.6%)
Type of admission	
Medical	38 (55.9%)
Surgical	14 (20.6%)
Trauma	16 (23.5%)
Primary diagnosis	
Cerebrovascular accident	22 (32.4%)
Sepsis	13 (19.1%)
Road traffic accident	11 (16.2%)
Chronic obstructive pulmonary disease	8 (11.8%)
Burns	5 (7.4%)
Ischaemic heart disease	3 (4.4%)
Other	6 (8.8%)

Most patients were admitted under medical services, which accounted for 38 cases (55.9%). Trauma-related admissions contributed 16 patients (23.5%), while surgical admissions made up the remaining 14 patients (20.6%). Cerebrovascular accident was the most common primary diagnosis, observed in 22 patients (32.4%), followed by sepsis in 13 patients (19.1%) and road traffic accident in 11 patients (16.2%). Chronic obstructive pulmonary disease was present in eight patients (11.8%), burn injuries in five patients (7.4%), and ischaemic heart disease in three patients (4.4%). Six patients (8.8%) had miscellaneous diagnoses grouped under the “Other” category.

Prior antibiotic exposure and risk factors

Prior antimicrobial exposure was common in this cohort. Fluoroquinolones had been administered in 11 patients (16.2%), whereas 46 patients (67.6%) had received a beta-lactam or carbapenem before developing VAP due to *Acinetobacter baumannii*. Only two patients (2.9%) had documented prior colonisation with methicillin-resistant *Staphylococcus aureus*. A wide range of additional antibiotics had been used, with colistin and amikacin being the most frequently prescribed agents, each administered in 13 patients (19.1%). Tigecycline was used in 12 patients (17.6%) and clindamycin in 11 patients (16.2%).

**Figure 1:**
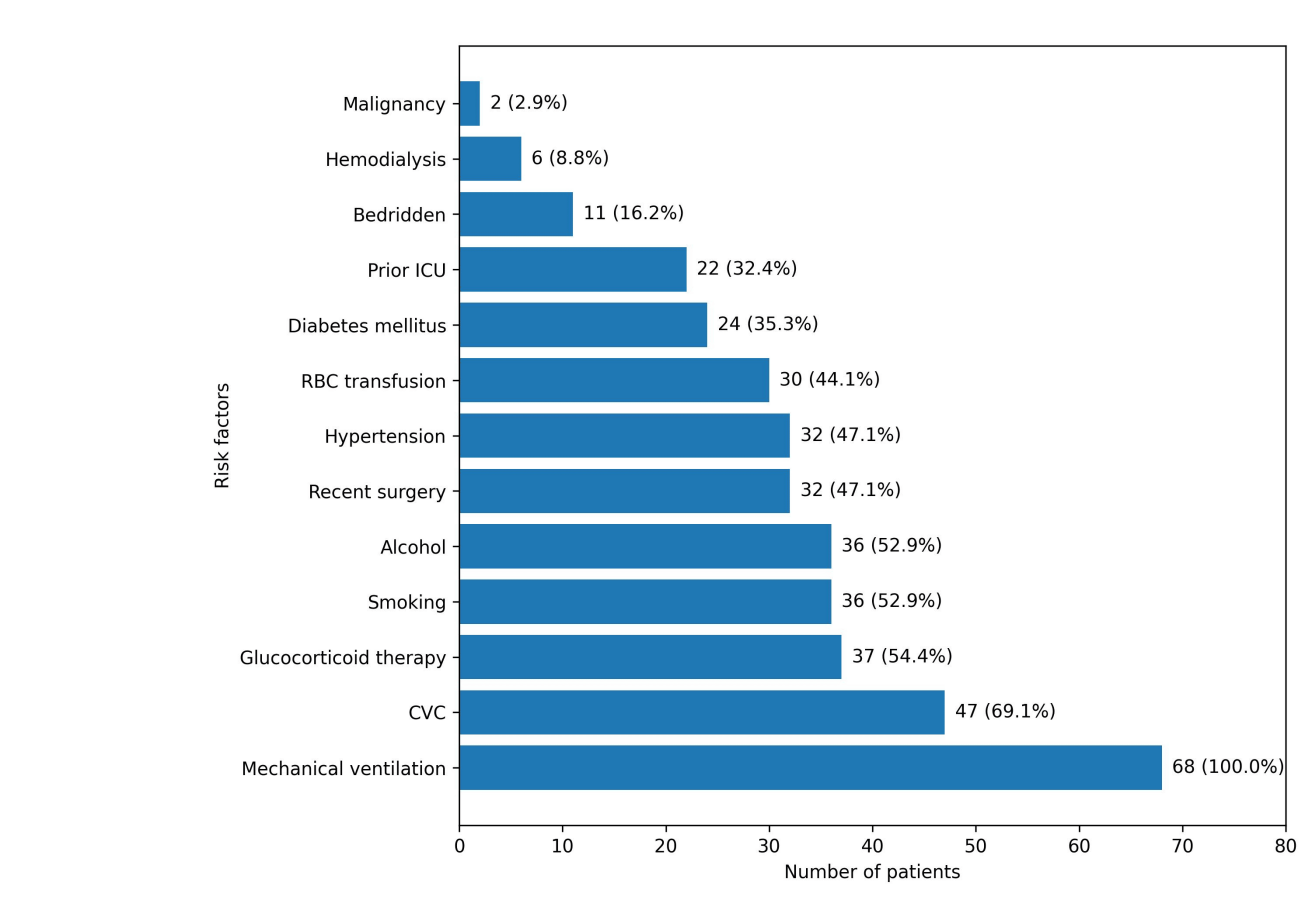
Distribution of major clinical risk factors among patients with ventilator-associated pneumonia due to Acinetobacter baumannii. Values are expressed as number of patients, with percentages shown in parentheses, n (%). Percentages were calculated using the total cohort size of 68. Multiple risk factors per patient were permitted.

Mechanical ventilation was universal and present in all patients. The most prevalent associated risk factor was central venous catheter use, documented in 47 patients (69.1%). Glucocorticoid therapy had been administered in 37 patients (54.4%). Smoking and alcohol use were each reported in 36 patients (52.9%). Recent surgery and hypertension were each present in 32 patients (47.1%), while 30 patients (44.1%) had received packed RBC transfusions. Diabetes mellitus was recorded in 24 patients (35.3%), and 22 patients (32.4%) had a history of prior ICU admission. Less frequent factors included bedridden status in 11 patients (16.2%), haemodialysis in 6 patients (8.8%), and malignancy in 2 patients (2.9%). The distribution of major risk factors is summarised in Figure 1.

Microbiological profile and antibiotic sensitivity

Endotracheal aspirates were the predominant source of isolates and yielded *Acinetobacter baumannii* in 61 patients (89.7%). Tracheostomy tube secretions accounted for 5 isolates (7.4%), whereas bronchoalveolar lavage and pleural fluid each contributed 1 isolate (1.5%).

Antimicrobial resistance was extensive. Pan-drug-resistant strains were identified in 49 isolates (72.1%), while 18 isolates (26.4%) demonstrated multidrug resistance. A single isolate (1.5%) was pan-sensitive. Individual drug sensitivity patterns showed limited activity across most antimicrobial classes. Colistin demonstrated the highest susceptibility in 11 isolates (16.2%), followed by tigecycline in 10 isolates (14.7%). The activity of beta-lactams, carbapenems, and aminoglycosides was minimal. The antibiotic sensitivity pattern is summarised in Table 2.

**Table 2:** Antibiotic sensitivity pattern of Acinetobacter baumannii isolates. Percentages represent the proportion of isolates demonstrating susceptibility to each antimicrobial agent. Pan-drug-resistant isolates were non-susceptible to all tested antimicrobial agents/classes within the institutional panel, whereas pan-sensitive isolates were susceptible to all tested antimicrobial agents.

Drug sensitivity pattern	n
Pan-drug resistant	49
Pan-sensitive	1
Colistin	11
Amikacin	2
Cefepime	3
Gentamicin	3
Levofloxacin	3
Trimethoprim-sulfamethoxazole	1
Tetracycline	5
Clindamycin	1
Linezolid	1
Tobramycin	3
Tigecycline	1
Ciprofloxacin	1
Meropenem	2
Cefotaxime	1

Hospital outcome

Hospital outcome data showed a high mortality burden in this cohort. A total of 44 patients (64.7%) died during their hospital stay, while 24 patients (35.3%) survived to discharge.

Multiple clinical and healthcare-related variables were significantly associated with mortality. Male sex was linked with higher death rates (32 patients (72.2%) among non-survivors vs nine patients (37.5%) among survivors), with an OR of 4.68 (p = 0.025). Prior exposure to beta-lactam or carbapenem antibiotics was also more common among those who died (35 patients (76.1%) vs 11 patients (45.8%)), corresponding to an OR of 4.60 (p = 0.007).

A previous ICU admission emerged as one of the strongest predictors of mortality. It was reported in 20 patients (45.5%) among non-survivors compared with only two patients (8.3%) among survivors (OR 9.17, p = 0.002). Similarly, the use of central venous catheters was significantly associated with increased mortality (37 patients (84.1%) vs 10 patients (41.7%); OR 7.40, p = 0.001). RBC transfusion showed a parallel pattern, recorded in 25 patients (56.8%) who died compared with five patients (20.8%) who were discharged (OR 5.00, p = 0.005).

Lifestyle-related factors demonstrated strong associations with outcome. Smoking was present in 32 patients (88.9%) among non-survivors compared with nine patients (37.5%) among survivors (OR 13.33, p < 0.001). Alcohol use showed a similar distribution (30 patients (83.3%) vs 11 patients (45.8%); OR 6.43, p = 0.001).

Among comorbidities, diabetes mellitus was more frequent in non-survivors (20 patients (83.3%) vs 13 patients (54.2%); OR 4.17, p = 0.020), while hypertension was also significantly associated with mortality (27 patients (84.4%) vs 11 patients (45.8%); OR 6.04, p = 0.002).

Other variables, including prior MRSA colonisation, bedridden status, recent surgery, haemodialysis, glucocorticoid therapy, and malignancy, did not show statistically significant associations with outcome. A complete summary of the risk factor distribution and effect estimates is presented in Table 3.

**Table 3:** Association of risk factors with hospital outcome. MRSA: Methicillin-resistant Staphylococcus aureus.

Risk factor	Death (N = 44), n (%)	Discharged (N = 24), n (%)	Odds ratio	95% CI	p-value
Sex (male)	39 (72.2%)	15 (27.8%)	4.68	1.348-16.251	0.025
Prior MRSA colonisation	1 (50.0%)	1 (50.0%)	0.535	0.032-8.953	1
Prior beta-lactam/carbapenem exposure	35 (76.1%)	11 (23.9%)	4.596	1.550-13.627	0.007
Bedridden status	8 (72.7%)	3 (27.3%)	1.556	0.372-6.512	0.734
Prior ICU admission	20 (90.9%)	2 (9.1%)	9.167	1.918-43.817	0.002
Central venous catheter use	37 (78.7%)	10 (21.3%)	7.4	2.355-23.255	0.001
Recent surgery	20 (62.5%)	12 (37.5%)	0.833	0.308-2.257	0.802
Mechanical ventilation	44 (100%)	24 (100%)	-	-	-
Haemodialysis	5 (83.3%)	1 (16.7%)	2.949	0.324-26.826	0.413
Malignancy	1 (50.0%)	1 (50.0%)	0.535	0.032-8.953	1
Glucocorticoid therapy	27 (73.0%)	10 (27.0%)	2.224	0.807-6.125	0.135
RBC transfusion	25 (83.3%)	5 (16.7%)	5	1.581-15.817	0.005
Smoking	32 (88.9%)	4 (11.1%)	13.333	3.775-47.099	<0.001
Alcohol use	30 (83.3%)	6 (16.7%)	6.429	2.096-19.718	0.001
Diabetes mellitus	20 (83.3%)	4 (16.7%)	4.167	1.222-14.207	0.02
Hypertension	27 (84.4%)	5 (15.6%)	6.035	1.898-19.195	0.002

Severity-of-illness scores demonstrated clear differences between survivors and non-survivors. Median GCS scores were lower among patients who died, and higher APACHE II, qSOFA, and SAPS II scores were observed in the non-survivor group. To assess the discriminative performance of these scores, ROC analysis was performed.

SAPS II showed the highest predictive accuracy for mortality, with an AUC of 0.971, followed by APACHE II (AUC 0.954), qSOFA (AUC 0.937), and GCS (AUC 0.928). SAPS II demonstrated a sensitivity of 42 patients (95.5%) and specificity of 22 patients (91.7%) at the optimal cut-off. A comparative summary of all scoring systems is presented in Table 4.

**Table 4:** Performance of severity scoring systems in predicting mortality. GCS: Glasgow Coma Scale; APACHE II: Acute Physiology and Chronic Health Evaluation II; qSOFA: quick Sequential Organ Failure Assessment; SAPS II: Simplified Acute Physiology Score II; AUC: Area under the curve; PPV: Positive predictive value; NPV: Negative predictive value.

Score	AUC	Sensitivity (%)	Specificity (%)	PPV (%)	NPV (%)	Accuracy (%)
GCS	0.928	86.4	83.3	90.5	76.9	85.3
APACHE II	0.954	88.6	87.5	92.9	80.8	88.2
qSOFA	0.937	97.7	79.2	89.6	95	91.2
SAPS II	0.971	95.5	91.7	95.5	91.7	94.1

## Discussion

VAP caused by *Acinetobacter baumannii *remains one of the most difficult ICU infections to manage, and the profile of patients in our cohort mirrors many of the patterns described in contemporary literature. In our study, men accounted for nearly four-fifths of all cases, yielding a male-to-female ratio of 3.9:1, with 54 patients (79.4%). This degree of male predominance is more marked than in the reports by Özgür ES et al., who observed 63% male involvement in extensively drug-resistant *A. baumannii* VAP [13], and by Inchai J et al., who reported 71% male patients in their 2015 series [14].

Cerebrovascular accident was the most common primary diagnosis in our dataset, accounting for 22 patients (32.4%). Neurological patients have consistently been identified as a high-risk group for *A. baumannii* VAP. Garnacho-Montero J et al. noted similar clustering of neurological cases within their MDR VAP cohort [11]. Esperatti M et al. also found that neurological diagnoses were disproportionately represented among ventilated ICU patients with nosocomial pneumonia [6]. Sepsis was present in 13 patients (19.1%), aligning with the 15-20% contribution reported by Rello J et al. [7] and Rosenthal VD et al. [3]. Road traffic accident accounted for 11 patients (16.2%), which is broadly comparable to prior ICU cohorts that included trauma among relevant underlying admission categories [14,21]. The clustering of cerebrovascular disease, sepsis, and trauma-related diagnoses in our cohort fits well within established global patterns.

Medical admissions accounted for 38 patients (55.9%) of cases, slightly higher than the 48% medical case distribution in the International Nosocomial Infection Control Consortium (INICC) global dataset [3], but similar to the range reported by Chawla R for Asian ICUs [4]. Surgical admissions, accounting for 14 patients (20.6%), fell within the 18-22% range described by Rello J et al. [7]. These parallels suggest that our ICU receives a case mix comparable to other large tertiary centres. Many of these observed patterns, including male predominance, neurological admissions, and high mortality, have been consistently reported in prior studies, suggesting that our findings largely align with established epidemiological trends.

Culture sources in our study were also comparable to international practice. Endotracheal aspirates yielded 61 isolates (89.7%), similar to the approximately 85% reported by Rello J et al. [7] and the 80-90% reported by Torres A et al. [22]. Tracheostomy-related isolates accounted for five isolates (7.4%), which also fell within the 5-10% range reported by Esperatti M et al. [6].

The antimicrobial resistance profile in our cohort reflects the severity of the regional resistance burden. Pan-drug-resistant isolates comprised 49 cultures (72.1%), almost identical to the approximately 70% pan-resistant prevalence summarised by Munoz-Price LS and Weinstein RA [23]. Multidrug-resistant isolates accounted for 18 cultures (26.4%), consistent with the 28% MDR rate reported by Kanafani ZA et al. [9]. Colistin susceptibility was observed in 11 isolates (16.2%), which is comparable to data in the current literature [24]. Tigecycline susceptibility at 10 isolates (14.7%) mirrors the activity ranges summarised by Maragakis LL and Perl TM for XDR *A. baumannii* [25]. Carbapenems, beta-lactams, and aminoglycosides showed minimal activity, consistent with the markedly reduced carbapenem susceptibility described by Doi Y et al. across multiple regions [26]. Overall, resistance patterns in this cohort mirror global observations but fall at the more severe end of the spectrum.

Prior antibiotic exposure was common. Prior beta-lactam or carbapenem exposure among 46 patients (67.6%) is comparable to the 60-70% pre-VAP exposure reported by Garnacho-Montero J et al. [11]. Fluoroquinolone exposure in 11 patients (16.2%) was similar to proportions reported by Kanafani ZA et al. [9], though slightly lower than the 25% described by Torres A et al. [22]. Prior exposure to colistin and amikacin, each seen in 13 patients (19.1%), corresponds to usage trends observed in ICUs managing multidrug-resistant Gram-negative infections.

Several clinical factors were found to be associated with mortality in our dataset. Central venous catheter use was significantly more frequent among non-survivors. Cunnion KM et al. reported CVC use in 72% of fatal nosocomial pneumonia cases [27], which is comparable to the proportions we observed. RBC transfusion also showed a strong association with mortality. Metheny NA et al. documented substantially higher mortality in transfused critically ill patients compared with those who were not transfused [28]. Smoking and alcohol use were each more prevalent among non-survivors, echoing findings by Papazian L et al., who reported higher mortality among patients with impaired pulmonary reserve [29]. Diabetes and hypertension also appeared more frequently among those who died, consistent with the comorbidity-linked vulnerability highlighted by Cook DJ et al. [21].

Organ dysfunction markers paralleled outcomes in this cohort. Non-survivors had higher creatinine and urea values, while mean haemoglobin was lower among non-survivors. These findings suggest that renal dysfunction and anaemia may reflect greater physiological severity in patients with poor outcomes, although causal inference is limited by the observational design.

Severity scoring systems showed strong associations with outcomes in this cohort. Non-survivors had APACHE II scores above 30, compared with scores near 12 among survivors. APACHE II thresholds above 20 have been associated with significantly increased mortality in VAP cohorts, as shown in analyses by Chastre J and Fagon JY [30]. SAPS II behaved similarly; earlier work by Le Gall JR et al. showed mortality approaching 80% at SAPS II thresholds above 40 [20], which is consistent with the outcomes in our high-score patients. The qSOFA performance in our cohort also aligned with its recognised prognostic value in critical illness literature [19]. However, these findings should be interpreted in the context of the observational design, absence of multivariable adjustment, and lack of external validation.

Overall mortality in our cohort was observed in 44 patients (64.7%). This is almost identical to the mortality reported by Garnacho-Montero J et al. for resistant *A. baumannii* VAP [11] and similar to the 63% mortality in the late-onset VAP group described by Kollef MH et al. [31]. Özgür ES et al. reported a mortality rate of 55% in their XDR cohort [13], and Inchai J et al. reported approximately 50% [14]. The slightly higher mortality in our study may be related to the high burden of antimicrobial resistance observed in this cohort. Our discharge rate of 24 patients (35.3%) also falls within the survival ranges described in international data [3,7,24].

Taken together, the findings from our ICU show a clinical and epidemiological profile consistent with global patterns of *A. baumannii* VAP but marked by very high resistance rates. The constellation of risk factors and outcomes follows established trends across multiple regions. By evaluating clinical characteristics, antimicrobial resistance patterns, and prognostic indicators together within a single cohort, this study provides a more integrated view of disease behaviour in a real-world ICU setting. The severe resistance profile may partly explain the high mortality observed in this cohort, although this cannot be confirmed without adjusted analysis.

Limitations

This study has several limitations that should be considered when interpreting the findings. The cross-sectional design limits the ability to establish causal relationships between identified clinical factors and outcomes. As a single-centre study conducted in a tertiary care intensive care unit, the findings may reflect local patient characteristics, antimicrobial prescribing practices, and resistance patterns, thereby limiting generalisability to other settings.

The relatively small sample size may have influenced the stability of the observed associations. In addition, the absence of multivariable analysis limits the ability to determine the independent effect of individual risk factors and raises the possibility of residual confounding. Continuous variables and severity scores were analysed descriptively, and their statistical handling may not fully account for underlying distributional differences.

The high discriminative performance of the severity scoring systems observed in this study should be interpreted in the context of the sample size and the lack of internal or external validation. ROC analysis was based on point estimates of AUC without interval estimation.

Microbiological analysis was based on routinely tested antibiotic panels, and molecular characterisation of resistance mechanisms was not performed. Resistance patterns were defined based on susceptibility results within the institutional antibiotic panel, which may differ from standardised classification systems.

## Conclusions

Patients who developed VAP due to *Acinetobacter baumannii* in our unit showed the familiar combination of prolonged ventilation, serious underlying illness, and prior exposure to broad-spectrum antibiotics. The high proportion of resistant isolates, especially the large group that was non-susceptible to all tested standard drugs, appears to have contributed to the outcomes observed in this cohort.

The overall picture from this study suggests that once severe physiological stress and organ dysfunction are established, recovery becomes difficult despite supportive care. Timely recognition of risk, faster access to microbiological results, and careful use of antibiotics may help limit the shift toward highly resistant disease. Consistent infection control practices, regular review of invasive devices, and close attention to ventilatory requirements remain practical steps that can reduce the burden of *A. baumannii* VAP in similar intensive care settings.

These findings should be interpreted in the context of the observational design and are best considered hypothesis-generating. Further multicentre studies with larger sample sizes and appropriate adjustment for confounding are required to validate these observations and define their clinical applicability.
